# Low self-recognition and awareness of past hypomanic and manic episodes in the general population

**DOI:** 10.1186/s40345-015-0039-8

**Published:** 2015-10-06

**Authors:** Eline J. Regeer, Ralph W. Kupka, Margreet ten Have, Wilma Vollebergh, Willem A. Nolen

**Affiliations:** Altrecht Institute for Mental Health Care, Nieuwe Houtenseweg 12, 3524 SH Utrecht, The Netherlands; Department of Psychiatry, VU Medical Center, Amsterdam, The Netherlands; Netherlands Institute of Mental Health and Addiction (Trimbos Institute), Utrecht, The Netherlands; Department of Social and Behavioural Sciences, Utrecht University, Utrecht, The Netherlands; Department of Psychiatry, University Medical Center, University of Groningen, Groningen, The Netherlands

**Keywords:** Bipolar disorder, Self-recognition, Awareness, General population

## Abstract

**Background:**

Bipolar disorder is often underdiagnosed and undertreated. Its detection and correct diagnosis highly relies on the report of past hypomanic or manic episodes. We investigated the recognition and awareness of past hypomanic and manic episodes in a sample of respondents with bipolar disorder selected from a general population study.

**Methods:**

In a reappraisal study from the Netherlands Mental Health Survey and Incidence Study (NEMESIS), we further investigated 40 respondents with lifetime bipolar disorder confirmed by the structured clinical interview for DSM-IV (SCID). Respondents were asked about awareness of past depressive, manic and hypomanic episodes, illness characteristics and treatment history.

**Results:**

Most respondents (82.5 %) recognized that they had experienced a depressive episode while 75 % had consulted a health professional for a depressive episode. Only a minority (22.5 %) recognized that they had experienced a (hypo)manic episode and only 17.5 % had consulted a health professional for a (hypo)manic episode. Only 12.5 % of the respondents reported having received a diagnosis of bipolar disorder. Recognition of previous (hypo)manic episodes was not related to severity of bipolar disorder.

**Conclusions:**

In routine clinical practice history-taking on a syndromal level, i.e., only inquiring whether a patient presenting with depression ever experienced a hypomanic or manic episode or received treatment for such an episode, is not sufficient to confirm or exclude a diagnosis of bipolar disorder. Other efforts, such as an interview with a significant other and the use of self report questionnaires or (semi-)structured interviews may be needed to recognize previous manic symptoms in patients with depression.

## Background

Population-based (Regier et al. 1993; Kessler et al. 1997; ten Have et al. 2002; Hirschfeld et al. 2003a; Wang et al. 2005; Schaffer et al. 2006; Merikangas et al. 2007) and clinical studies (Manning et al. 1997; Hantouche et al. 1998; Ghaemi et al. 1999, 2000; Mantere et al. 2004; Das et al. 2005; Hirschfeld et al. 2005; Smith et al. 2011; Rogers et al. 2012; Inoue et al. 2015) report that bipolar disorder (BD) is often underdiagnosed and undertreated. A late diagnosis of BD is partly inevitable since in most patients with BD (BD patients) depressive episodes precede a first (hypo)manic episode. However, retrospective studies among BD patients report also long delays between onset of the first manic/hypomanic episode and receiving a correct diagnosis and the start of adequate treatment (Lish et al. 1994; Suppes et al. 2001; Hirschfeld et al. 2003b; Morselli and Elgie 2003; Berk et al. 2007; Drancourt et al. 2013). Since most BD patients will present with depressive symptoms (Kupka et al. 2007), making a correct diagnosis highly relies on the report of past (hypo)manic episodes. Patients must be aware that some past behaviors were actually symptoms of a (hypo)manic episode. Moreover, if these past symptoms/episodes have been recognized, this diagnosis must be remembered, indicating that the patient is aware and acknowledges that he is suffering from BD.

Previously we performed a reappraisal study with the Structured Clinical Interview for DSM-IV axis I (SCID-I) (Spitzer et al. 1992) among all respondents from the Netherlands Mental Health Survey and Incidence Study (NEMESIS) who were diagnosed with DSM-III-R BD using the Composite International Diagnostic Interview 1.1 (CIDI 1.1). It can be assumed that a semi-structured interview, such as the SCID, with the flexible open-ended and conversational probing by clinicians will have more concordance with clinical diagnoses than diagnoses based on fully structured interviews with yes or no answers administered by lay interviewers, such as the CIDI. In a first paper resulting from that study, we published on the prevalence of bipolar disorder in the general population based on the SCID and the possible explanations for discrepancies between the CIDI and SCID diagnoses (Regeer et al. 2004).

For the present report, we investigated the recognition and awareness of past depressive and (hypo)manic episodes in a sample of respondents selected from this study in the general population, thus avoiding the bias of help-seeking behavior as would be the case in clinical populations. We examined whether the respondents identified with lifetime SCID/DSM-IV diagnosis of BD were aware and acknowledged that the depressive and manic symptoms they reported during the SCID interview occurred as part of a depressive or (hypo)manic episode, respectively.

We hypothesized a priori that most respondents with depressive symptoms would have seeked help and agreed with the clinician that these symptoms are part of a depressive episode while in contrast most people with manic symptoms, would not have recognized these as being part of a (hypo)manic episode. We further hypothesized that respondents with bipolar I disorder (BD-I) would report past (hypo)manic episodes more often than respondents with bipolar II disorder (BD-II), BD NOS, or cyclothymia and that those respondents who acknowledged that they had suffered from (hypo)manic episodes would have experienced more manic symptoms, reflecting more severe episodes. Finally, we hypothesized that respondents who were aware of past (hypo)manic episodes and who recalled and reported a former diagnosis of BD would more frequently have received adequate treatment.

## Methods

### Study sample

Respondents for this study were selected from NEMESIS, a prospective study in the Dutch general population (*N* = 7076) aged 18–64, with three assessment points (baseline, *T*_1_ and *T*_2_) in 1996, 1997 and 1999. The respondents reflect the Dutch population in terms of gender, civil status, and urbanity, with the exception of a slight underrepresentation of individuals in the age group between 18 and 24 years (Bijl et al. 1998a, b).

Of all 158 respondents who were identified with a lifetime CIDI/DSM-III-R BD at any of the three assessment points in NEMESIS 105 indicated that they could be contacted in case of follow-up studies. Ultimately 74 of the 105 respondents (70.5 %) participated in the reappraisal study, i.e. 46.8 % of the total sample with a CIDI-diagnosed bipolar disorder. These participants (*N* = 74) were significantly older, higher educated and more employed than the non-participants (*N* = 84). There were no significant differences regarding gender, household composition, urbanicity, income, comorbidity and prevalence of bipolar I disorder versus bipolar disorder NOS.

In order to keep the interviewers blind for the original CIDI/DSM-III-R diagnosis a second group of 57 NEMESIS-respondents with a lifetime diagnosis of major depressive disorder (MDD) was randomly selected of whom 40 (70 %) participated in the present study. No significant differences between participants and non-participants were found regarding sociodemographic factors and comorbidity.

**Fig. 1:**
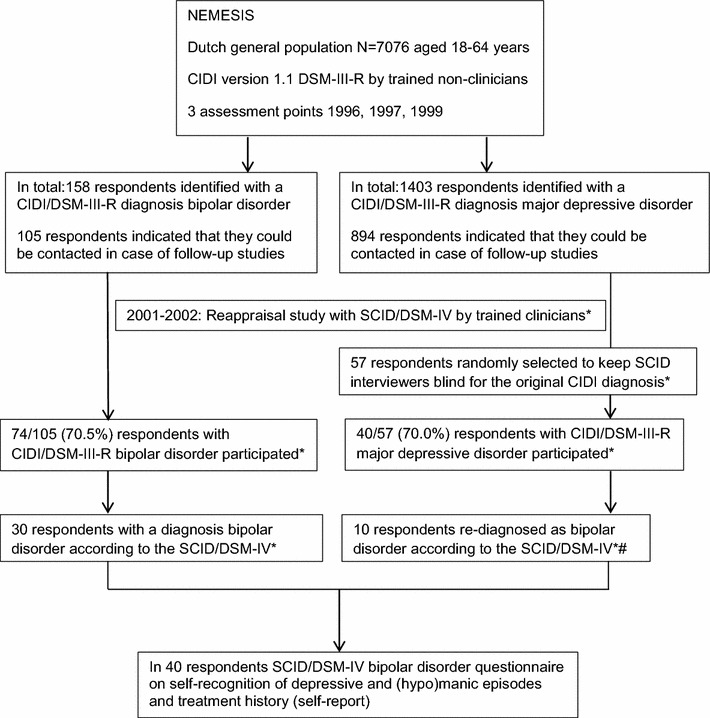
Recruitment and respondent flow, the diagnostic instruments used and whether information came from an interview or self-report. *For further details on the method see the previously published paper (Regeer et al. 2004). # Including two respondents who developed the first (hypo)manic episode between the last CIDI interview and the SCID interview

Thirty of the 74 respondents with a CIDI/DSM-III-R BD and 10 of the 40 respondents with a CIDI/DSM-III-R MDD met the DSM-IV criteria for bipolar disorder when interviewed with the SCID. Thus, we identified 40 respondents with a lifetime SCID/DSM-IV diagnosis of BD (see Fig. 1).

All respondents for the present study were interviewed at home between August 2001 and February 2002. Written informed consent was obtained from all respondents. The study was approved by the Medical Review Board of the University Medical Centre in Utrecht.

### Diagnosis of bipolar disorder

All respondents were assessed with the SCID-I (Spitzer et al. 1992) for current and lifetime DSM-IV axis I diagnoses, subtype of BD and the number of symptoms experienced during the mood episodes. The most severe current (previous month) and past (hypo)manic and depressive episodes were explored. To diagnose a (hypo)manic episode we adhered to the DSM-IV inclusion and exclusion criteria, for type, number and duration of symptoms. After completing the SCID interview all respondents were informed about the identified diagnoses.

### Self-recognition of depressive and (hypo)manic episodes

Respondents of whom a diagnosis of BD was confirmed by the SCID-I were asked about recognition of this diagnosis by the following questions: “Do you recognize that you have had depressive/(hypo)manic episodes?” In case participants did not recognize previous depressive or (hypo)manic episodes they were asked: “What is your explanation for the symptoms you mentioned during the interview?” (see “Appendix 1”).

### Past diagnosis and treatment history

Subsequently, treatment history was examined by the following questions: “Did you consult a health professional for depressive episodes? Which diagnosis did you receive when seeking help for your depressive episodes? Did you consult a health professional for (hypo)manic episodes? Which diagnosis did you receive when seeking help for (hypo)manic episodes? Did you ever receive a diagnosis of bipolar disorder? Which treatment did you receive (psycho-education, psychotherapy, medication (mood stabilizers, antipsychotics, anti-anxiety drugs, hypnotics), hospitalization for a manic or depressive episode, or another treatment)?” (see “Appendix 1”). Respondents were considered to have received minimally adequate treatment for bipolar disorder if they had ever used a mood stabilizer (lithium, carbamazepine or valproate) and had received treatment from a psychiatrist or psychologist.

### Statistics

Analyses were carried out with SPSS 20.0 for Windows. We used summary statistics to describe the sample. Data were analyzed using standard cross tabulation. Fisher’s Exact test of significance (2-sided) was used to compare percentages between respondents with bipolar I disorder and bipolar spectrum disorder. A significant level of *P* < 0.05 was used.

## Results

The re-appraised sample consisted of 14 respondents with BD-I, 14 with BD-II, 7 with BD NOS, 3 with cyclothymia and 2 with BD induced by an antidepressant. For the analyses the 26 respondents with non-bipolar I disorder were grouped as bipolar spectrum disorder (BD spectrum).

The mean age of respondents was 43.8 years, and 62.5 % was female. The mean number of depressive symptoms experienced during the most severe depressive episode was 6.1 (95 % CI 5.3–6.8). The mean number of manic symptoms experienced during the most severe hypomanic/manic episode was 6.3 (95 % CI 5.6–6.9). Respondents with BD-I had experienced a significantly higher mean number of symptoms during the most severe (hypo)manic episode than respondents with BD spectrum (7.4 (95 % CI 6.4–8.3) vs 5.7 (95 % CI 5.0–6.5) *P* = 0.009). The rates of lifetime manic symptoms are shown in Table 1. The most reported manic symptoms are elevated mood (*n* = 39, 97.5 %), increased activity (*n* = 34, 85.0 %) and more talkative (*n* = 29, 72.5 %). No significant differences were found in the rates of manic symptoms between respondents with BD-I and BD spectrum, with the exception of involvement in activities with high potential for painful consequences. Respondents with BD-I reported this symptom more often than respondents with BD spectrum (*n* = 8, 57.1 % vs *n* = 3, 11.5 %, *P* = 0.007).

**Table 1:** Presence of lifetime manic symptoms in 40 respondents with a SCID/DSM-IV diagnosis bipolar disorder

	Bipolar I disorder (*N* = 14) *N* (%)	Bipolar spectrum disorder (*N* = 26) *N* (%)	Bipolar disorder total (*N* = 40) N (%)	Significance Fisher exact test
Elevated mood	13 (92.9)	26 (100)	39 (97.5)	ns
Increased activity	11 (78.6)	23 (88.5)	34 (85.0)	ns
More talkative	13 (92.9)	16 (61.5)	29 (72.5)	ns
Inflated self-esteem/grandiosity	11 (78.6)	17 (65.4)	28 (70.0)	ns
Flight of ideas/racing thoughts	11 (78.6)	17 (65.4)	28 (70.0)	ns
Distractibility	11 (78.6)	17 (65.4)	28 (70.0)	ns
Decreased need for sleep	11 (78.6)	15 (57.7)	26 (65.0)	ns
Psychomotor agitation	9 (64.3)	8 (30.8)	17 (42.5)	ns
Irritable mood	5 (35.7)	6 (23.1)	11 (27.5)	ns
Involvement in activities with high potential for painful consequences	8 (57.1)	3 (11.5)	11 (27.5)	*P* = 0.007
Mean number of symptoms	7.4	5.7	6.3	*P* = 0.009

*ns* Non significant difference between both groups

Most respondents (*n* = 33, 82.5 %) recognized that they had suffered from depressive episodes (Table 2) and 75.0 % (*n* = 30) had consulted a health professional for these episodes. Only 9 out of 40 (22.5 %) recognized that they had experienced (hypo)manic episodes in the past. We found no difference between respondents with BD-I (*n* = 3, 21.4 %) and BD spectrum (*n* = 6, 23.1 %). Only 7 (17.5 %) had consulted a health professional for (hypo)manic episodes: significantly more respondents with BD-I (*n* = 5, 35.7 %) than with BD spectrum (*n* = 2, 7.7 %) (*P* = 0.039). Only 5 of 40 (12.5 %) indicated that they previously had received a diagnosis of BD, had used a mood stabilizer and had received psychiatric treatment. Significantly more respondents with BD-I than with BD spectrum reported a past diagnosis of BD (respectively, *n* = 4, 28.6 % vs *n* = 1, 3.8 %; *P* = 0.043) and past hospitalization for a (hypo)manic episode (*n* = 7, 50.0 % vs *n* = 0; 0.0 %; *P* = 0.000).

**Table 2:** Self-recognition of mood episodes, consultation of health professionals, hospitalization, self-reported diagnosis of bipolar disorder and treatment in 40 respondents with a SCID/DSM-IV diagnosis bipolar disorder

	Bipolar I disorder (*N* = 14) *N* (%)	Bipolar spectrum disorder (*N* = 26) *N* (%)	Bipolar disorder total (*N* = 40) *N* (%)	Significance Fisher exact test
Recognized past depressive episodes	11 (78.6)	22 (84.6)	33 (82.5)	ns
Recognized past (hypo)manic episodes	3 (21.4)	6 (23.1)	9 (22.5)	ns
Consulted health professional for depressive episodes	11 (78.6)	19 (73.1)	30 (75.0)	ns
Consulted health professional for (hypo)manic episodes	5 (35.7)	2 (7.7)	7 (17.5)	*P* = 0.039
Hospitalization for a manic episode	7 (50)	0 (0)	7 (17.5)	*P* = 0.000
Indicated a past diagnosis of bipolar disorder	4 (28.6)	1 (3.8)	5 (12.5)	*P* = 0.043
Treatment for bipolar disorder	4 (28.6)	1 (3.8)	5 (12.5)	*P* = 0.043

*ns* Non significant difference between both groups

Respondents who recognized that they had suffered from (hypo)manic episodes did not experience a significantly higher number of manic symptoms than respondents who did not (not presented in Table 2: 7.1 vs 6.0, *P* = ns). The rates of lifetime manic symptoms did not differ significantly between respondents who recognized that they had suffered from (hypo)manic episodes and respondents who did not (data not shown).The 31 respondents who did not recognize that past manic symptoms were part of a (hypo)manic episode gave the following explanations: these phenomena are pleasant and productive (*N* = 14), are part of my personality (*N* = 5), were the results of circumstances (post-partum, trauma, death of a significant other) (*N* = 5), were diagnosed as psychosis (*N* = 2), were the effect of treatment with an antidepressant (*N* = 2), or other explanations (*N* = 3).

## Discussion

Our major finding is that self-recognition of past (hypo)manic episodes among subjects selected from the general population and diagnosed with a lifetime SCID diagnosis of bipolar I disorder or bipolar spectrum disorder is low. Only 22.5 % recognized that they had experienced a (hypo)manic episode, only 17.5 % had consulted a health professional for a (hypo)manic episode, and only 12.5 % remembered having received a diagnosis of bipolar disorder and had received minimally adequate treatment. In contrast, most respondents (82.5 %) recognized that they had experienced a depressive episode and 75 % had consulted a health professional for a depressive episode. In contrast to our hypothesis, respondents with BD-I did not recognize previous manic episodes more often than respondents with BD spectrum. In addition, respondents who recognized that they had experienced (hypo)manic episodes did not report a greater number of manic symptoms than those who did not recognize that they had experienced (hypo)manic episodes. Interestingly, most respondents in our study (85 %) reported increased activity during a (hypo)manic episode. This confirms the inclusion of increased activity or energy level as a core symptom for a (hypo)manic in the DSM-V (American Psychiatric Association 2013).

It is well known that hypomania in BD-II may not be recognized by patients as part of their mood disorder, being often perceived as ego-syntonic and associated with better social and occupational function. The results of our study suggest that even in the case of BD-I, recognition and awareness of past manic episodes is low. Respondents gave alternative explanations for their manic symptoms, explaining them as pleasant and productive periods, part of their personality, and as a result of various psychosocial circumstances. Although manic episodes can be severe and by definition are associated with negative consequences, they are also associated with low awareness. Hence, reliance on patient self-report contributes to the underdiagnosis of mania (Ghaemi et al. 2002).

Another factor that could influence self-recognition is a state-dependent memory distortion, i.e., difficulties in remembering (hypo)manic episodes during a depressive state (Akiskal et al. 2000). However, in our general population study most respondents were euthymic during the interview and therefore state-dependent memory disturbances presumably did not influence recall.

In conclusion, when specifically asked in the context of a structured interview, respondents reported (hypo)mania at a symptomatic level, but when subsequently asked on a syndromal level most of them did not recognize that they had experienced a (hypo)manic episode. Past overactivity, euphoria or irritability were not perceived as pathological phenomena and were often experienced as ego-syntonic. This possibly explains the low percentage of respondents who consulted a health professional for these symptoms.

Our findings suggest that in clinical practice history-taking on a syndromal level, i.e., inquiring whether a patient who presents with depression ever experienced a (hypo)manic episode or received treatment for such an episode, is not sufficient to confirm or exclude a diagnosis of BD. Therefore, it is necessary to explore (hypo)mania on a symptomatic level. In our study we used a (semi)structured interview such as the SCID, which may be too extensive to be used in clinical practice. An alternative option is to rely on self-report screening questionnaires such as the Mood Disorder Questionnaire (MDQ, Hirschfeld et al. 2003c) or the hypomania checklist (HCL-32, Angst et al. 2005). Several studies have indicated that these can detect at least a proportion of the until then unrecognized patients (Das et al. 2005; Hirschfeld et al. 2005; Forty et al. 2009, Zimmerman and Galione 2011; Smith et al. 2011; Hu et al. 2012; Boschloo et al. 2013; Wang et al. 2015; Carvalho et al. 2015). As demonstrated by Angst et al. (2011) strong indicators of bipolarity in patients presenting with depression are: family history of hypomania/mania, early onset, recurrence of mood episodes, comorbid substance use disorder, and antidepressant-induced (hypo)mania. In addition, interviewing a significant other may be indispensable in the diagnostic process of bipolar disorder. Our results furthermore stress the importance of psycho-education once bipolar disorder has been diagnosed, especially on the nature of manic symptoms to improve awareness. Studies have shown positive effects of psycho-education and psychological treatment (i.e., cognitive behavioral therapy, interpersonal social rhythm therapy and family-focused therapy) on the acceptance of the diagnosis and medication adherence (Colom and Lam 2005; Miklowitz et al. 2007; Miklowitz and Scott 2009, Reinares et al. 2014). In addition, retrospective and prospective mood charting could be helpful in improving insight into the course of illness and adherence to therapy (Baldassano 2005).

## Limitations

A limitation of our study is the small number of participants. This is inevitable when respondents are recruited from the general population study and the prevalence of the disorder is low.

However, we are not aware of any study examining self-recognition of manic and hypomanic episodes and treatment history of bipolar disorder in relationship to formal DSM-IV diagnoses in general population subjects (re-)interviewed by clinicians. These patients are likely to consult a general physician or psychologist for depressive symptoms, at which point a history of mania or hypomania and hence a diagnosis of bipolar disorder may easily be missed if not assessed at a symptomatic level.

## Appendix 1: Questionnaire

In respondents identified with Bipolar Disorder based on the SCID:

- Do they recognize themselves that they have or have had depressive episodes and (hypo)manic episodes, respectively?
  - If they do not recognize it themselves: what are their explanations for their problems/symptoms?
- When did they have their first depressive episode?
  - When did they for the first time consult a (mental) health professional for this (or later) episode; and which diagnosis was made; if not: why not?
- When did they have their first (hypo)manic episode?
  - When did they for the first time consult a (mental) health professional for this (or later) episode; and which diagnosis was made; if not: why not?
- When (at what age) was the diagnosis of BPD made for the first time?
- Did they ever receive treatment? If yes: when (at what age) for the first time:
  - Psycho-education.
  - Psychotherapy.
  - Medication: (separately for mood stabilizers, antipsychotics, antidepressants, anti-anxiety drugs (benzodiazepines), hypnotics).
  - Other treatments.
